# The experience of trial participation, treatment approaches and perceptions of change among participants with dissociative seizures within the CODES randomized controlled trial: A qualitative study

**DOI:** 10.1016/j.yebeh.2020.107230

**Published:** 2020-10

**Authors:** Julie Read, Harriet Jordan, Iain Perdue, James Purnell, Joanna Murray, Trudie Chalder, Markus Reuber, Jon Stone, Laura H. Goldstein

**Affiliations:** a King's College London, Institute of Psychiatry, Psychology and Neuroscience, London, UK; b Academic Neurology Unit, University of Sheffield, Royal Hallamshire Hospital, Sheffield S10 2JF, UK; c Department of Clinical Neuroscience, Centre for Clinical Brain Sciences, University of Edinburgh, UK

**Keywords:** Dissociative seizures, Cognitive behavior therapy, Seizure control, Change, Qualitative

## Abstract

**Background:**

Nested within a large, multicenter randomized controlled trial (RCT) for people with dissociative seizures (DS), the study used purposive sampling to explore participants' experience of participating in an RCT, their experience of DS-specific cognitive behavioral therapy (CBT) and another component of the RCT, Standardized Medical Care (SMC) and their perceptions of and reflections on seizure management and change.

**Methods:**

A qualitative study using semistructured interviews was conducted with 30 participants in an RCT (the COgnitive behavioral therapy vs standardized medical care for adults with Dissociative non-Epileptic Seizures (CODES) Trial) investigating the effectiveness of two treatments for DS. Key themes and subthemes were identified using thematic framework analysis (TFA).

**Results:**

Analysis yielded three overarching themes: taking part in a treatment trial — “the only thing out there”, the experience of treatment techniques that were perceived to help with seizure management, and reflections on an “unpredictable recovery”.

**Conclusions:**

People with DS are amenable to participating in a psychotherapy RCT and described a largely positive experience. They also described the applicability of aspects of DS-specific CBT and SMC in the management of their DS, received within the confines of the CODES trial. Factors that appeared to account for the variability in response to treatment delivery included individual preferences for the nature of sessions, the nature of therapeutic relationships, readiness to discuss trauma, other aspects of emotional avoidance, and whether therapy provided something new.

## Highlights

• Participants generally perceived participating in an RCT as a positive experience.
• Participants perceived that the CODES RCT offered them the possibility of change.
• Breathing and distraction techniques were perceived to help with seizure management.
• Participants identified the importance of a good therapeutic alliance.
• Seizure control or acceptance of ongoing seizures was perceived as progress.

## 1 Introduction

Dissociative seizures (DS) are paroxysmal events that resemble epileptic seizures or syncope but are not associated with abnormal brain electrical activity that would indicate epilepsy and are not explained by other medical conditions. They are understood as a dissociative response to potentially provoking internal or external stimuli [1]. Dissociative seizures are more common in women and are often initially misdiagnosed as epilepsy or syncope while the correct diagnosis is commonly delayed by several years [2]. DS are associated with high rates of comorbid psychopathology [3,4] as well as other functional neurological or somatic symptoms [5]. Dissociative seizures and epilepsy can also co-occur [6]. Importantly, it has been noted that previous psychological trauma, while common, is not essential for the diagnosis [4].

Recent research into the phenomenology and semiology of DS has led to the development of biopsychosocial models of the condition. While a variety of factors lead to the maintenance of DS [7], fear and avoidance may be conceived as particularly important targets for therapy since they may lead to a wide range of everyday activities being altered or avoided [8], consistent with the fear-escape avoidance model [9]. One approach using cognitive behavioral therapy (CBT) has indeed built upon the fear-avoidance model leading to a series of treatment studies [8,10,11] and culminating in the first sufficiently powered randomized controlled trial (RCT) to compare treatments for patients with DS, i.e., the COgnitive behavioral therapy vs standardized medical care for adults with Dissociative non-Epileptic Seizures (CODES) trial [12].

Previous qualitative research with patients with DS is summarized well in Rawlings and Reuber's [13] systematic synthesis that included 21 studies from the previous 20 years. Characteristic of these studies was a small sample size of 5–15 participants. Many of these studies noted the crippling isolation and debilitating effects of long-term DS. One study examining participants' perceptions of DS after a psychoeducational program, reported mixed results in terms of perceptions of improvement [14]. Fairclough et al. [15] explored patients' concerns and understanding prior to psychological treatment and found apprehension about whether psychological treatment would meet their needs. Other studies focused on describing the heterogeneous nature of DS disorders, problems surrounding the differential diagnosis, the burden of DS, and difficulties surrounding emotional processing and treatments [13,16].

There is a paucity of research on patients with Functional Neurological Disorder (FND), much less DS, participating in an RCT involving a complex intervention. This paper seeks to address the knowledge gap about the experience of people with DS taking part in an RCT more generally and their experience of the specific interventions within the CODES RCT. We wanted to know more about the techniques or treatment in both arms, which were perceived by participants to bring about change. Undertaking qualitative work of this nature can help shed light on why treatment might be successful but also what might explain treatment failure.

## 2 Materials and methods

### 2.1 Interventions and outcomes in the CODES study

In the CODES RCT, 368 individuals with DS were randomized to receive Standardized Medical Care (SMC) alone or DS-specific CBT in addition to SMC (CBT + SMC) [12].

Standardized Medical Care involved a formalized approach to the delivery of the diagnosis at the neurology/specialist epilepsy clinic and two different information factsheets — one provided by neurologists following initial diagnosis and one by psychiatrists at the subsequent neuropsychiatric assessment (see http://www.codestrial.org/information-booklets/4579871164). Following the diagnosis by a neurologist, where the diagnosis and rationale for referring the patient to a psychiatrist were explained [12], around three months later liaison or neuropsychiatrists carried out further assessments of etiological and maintaining factors, treated complex comorbidity, offered psychopharmacological interventions (as appropriate), and gave advice on seizure management but were instructed to avoid using CBT techniques. Standardized Medical Care follow-up appointments were primarily offered by the CODES psychiatrist but also, to a lesser extent, by the CODES diagnosing neurologist. We suggested that neurology SMC follow-up sessions might review progress and the patient's ongoing understanding of their diagnosis, oversee withdrawal of antiepileptic drugs if appropriate, provide management for comorbid physical conditions, and reassess where appropriate major psychiatric risk; neurologists could also institute psychopharmacological treatments for anxiety or depression prior to the psychiatric assessment in cases of clinical need and could help with the completion of any government department forms e.g., concerning driving. As part of the psychiatrists' input [12], there was the opportunity for them to provide general information about any warning symptoms concerning DS and distraction, but the intention was that particular techniques would not be covered so that this did not become therapy as such. Psychiatrists were also able to liaise with other mental health professionals involved in the person's care and refer to crisis teams if necessary, but referrals for other psychotherapy, specifically for DS, was not to be made; it was intended that psychiatrists would manage patients' symptoms in the usual way. While we did not prescribe the number of SMC sessions to be offered, since these may have been influenced both by local service availability and also patients' clinical need, we anticipated that there might be up to two SMC sessions offered by neurologists following diagnosis delivery and three to four SMC sessions offered by psychiatrists [12].

Those randomized to CBT in addition to SMC (CBT + SMC) were offered a DS-specific CBT intervention comprising of 12 one-hour-long sessions intended to occur over four to five months delivered by qualified CBT therapists, with a further booster session nine months after randomization. Therapy was manualized with guidance for each session but was individualized and formulation-driven and allowed flexibility in treatment delivery. In addition to engaging participants and providing psychoeducation — in particular teaching techniques to assist with seizure control — the intervention addressed avoidance behavior, identifying and challenging seizure-related cognitions, addressing trauma where relevant, and devising a plan for relapse prevention [17]. Therapists received training in delivering the DS-specific CBT by the CODES team and were supervised during the trial. Patients were provided with their own handbook containing supporting material for the intervention. Because of random allocation to treatment arms within an RCT, all patients randomized after receiving the diagnosis from a neurologist and undergoing prerandomization assessment by a psychiatrist were considered eligible for CBT in the study, unlike in routine clinical practice where patients might undergo further screening of their suitability for treatment and motivation to change by a therapist prior to commencement of CBT.

The primary outcome of the RCT was monthly DS frequency evaluated at 12 months postrandomization. Secondary outcomes included other measures related to DS occurrence including participants' ratings of how severe and bothersome they found their DS, the longest number of consecutive days without DS in the last six months of their time in the study, whether or not they were completely seizure-free in the final three months of the study, and whether or not their DS frequency reduced by more than 50%. Other secondary outcomes evaluated at 12 months covered a range of measures of psychological and psychosocial status including self-rated quality of life, depression, anxiety, distress, numbers of somatic symptoms, psychosocial functioning, and clinician and self-rated improvement as well as satisfaction with treatment. More details are provided elsewhere [12].

### 2.2 Participants

Purposive sampling was carried out with consideration of matching age, gender, ethnicity, and geographic characteristics of the sample in the broader RCT and was used to select 30 participants who had recently completed the CODES trial. They had previously consented to being contacted for an interview once they had finished the final follow-up assessment, a year following randomization. There was a deliberate weighting towards participants who had received the DS-specific CBT given the need to understand more about participants' experience of this intervention. We also selected some participants who were ambivalent about their treatment or who did not engage with the full course of treatment, to enable us to capture a range of views. Eligibility criteria for the CODES Trial are described elsewhere [12] but included patients with a previous diagnosis of epilepsy but who had not experienced an epileptic seizure in the previous year at the time of consent to the RCT. Twenty-one women and nine men were interviewed by researchers from the CODES team. A further two people were approached but declined to take part. All those participating gave written informed consent; consent was reconfirmed at the time of the interview. The CODES study received ethical approval from the London - Camberwell St Giles Research Ethics Committee (reference 13/LO/1595).

### 2.3 Interview schedule

The interview schedule (see Supplementary Material 1) was developed by several researchers in the CODES team to ensure clinical relevance and methodological rigor. The schedule was also discussed with a Service User (i.e., a patient representative) involved in the wider CODES project. The topics of relevance to the current paper were a) the experience of taking part in the CODES trial, b) their views on the treatment to which they were assigned, and c) their perception of treatment outcome. The remaining topics that were explored (participants' experience of receiving the diagnosis and the impact of DS on their life) will be reported elsewhere. A mid-way review of the interview schedule resulted in the addition of a further question that asked whether any reduction in DS had been accompanied by increased anxiety or depression. This addition arose because two participants interviewed by that point had said that this had been their experience. Positive as well as negative reflections were encouraged.

### 2.4 Data collection

Between February 2016 and May 2018, three researchers conducted the semistructured interviews in England and Scotland; one researcher (JR) conducted and transcribed most of the interviews. Twenty-five participants were interviewed at home, four participants from one NHS Trust were interviewed in a local hospital, and one participant requested the interview be at the CODES research worker's university offices. Interviews lasted between 45 and 90 min and were recorded on an encrypted digital voice recorder. Three of the interviewees had one family member or partner present.

### 2.5 Data analysis

Four members of the research team contributed to the verbatim transcription of the interviews. Participants' nonverbal cues were not included. Participant pseudoanonymity was ensured both in the recording and in the transcripts by using ID numbers. Healthcare professionals named in the recordings were anonymized in the transcriptions.

We adopted thematic framework analysis (TFA) [18], which is viewed as a useful approach to the analysis of qualitative data when a multidisciplinary team of researchers is involved in conducting qualitative research, and while initially devised for large-scale policy research, it is also used for health research [19] especially where research sets out to obtain answers to specific problems. Thus, the study's objectives must clearly relate to what is being asked about [18]. The approach is appropriate for use in mixed-methods research projects with multiple researchers [19,20]. Unlike Grounded Theory, TFA is not used to develop theory and is not linked to a particular theoretical, philosophical, or epistemological approach [19].

The qualitative analysis employed here comprised five stages.

1) The lead researcher (JR) initially read and re-read the transcripts in a process of familiarization with the data. Using NVivo v12 software (QSR International), each line of the transcripts was read and coded by the lead researcher (JR) in both a deductive way (in line with the a priori aims of the study) and then in an inductive way with multiple open codes. A second researcher (HJ) followed the same procedure and coded a total of eight randomly chosen transcripts relating to interviews from both trial arms. Coding meetings between the two researchers and the other members of the research team showed considerable agreement in coding and in the definition of themes arising from the grouping of codes into categories. 2) A working theoretical framework was developed from these categories. 3) This framework was then applied back to all subsequent transcripts for indexing to see how the raw data fitted this framework. 4) Adjustments to the framework were made as necessary. A matrix was developed on NVivo, and excerpts of raw data were presented in a chart to illustrate a given category [18]. 5) In a final stage – ‘*mapping and interpretation’* [21] – the lead researcher considered the connections and patterns between the charted categories and any explanations that might account for these connections.

## 3 Results

### 3.1 Participants

Twenty-two patients allocated to CBT + SMC and eight patients allocated to SMC were interviewed. See Table 1 for participant characteristics.

**Table 1:** Demographics of participants.

Demographic characteristic	N	%
*Age at interview (years)*		
18–30	10	33.3
31–40	6	20
41–50	7	23.3
51–60	2	6.6
61–80	5	16.6

*Gender*		
Female	21	70
Male	9	30

*Ethnicity*		
White British	28	93.3
Other	2	6.7

*Treatment received*		
CBT + SMC	22	73
SMC alone	8	27

*Number of CBT sessions attended*		
12 sessions + booster session	14	63.6
12 sessions	1	4.5
11 sessions	3	13.6
10 sessions	1	4.5
9 sessions	1	4.5
5 sessions	1	4.5
1 session	1	4.5

*Duration of DS*		
< 1 year	5	16.7
1–4 years	11	36.7
5–9 years	4	13.3
10–14 years	4	13.3
15–19 years	2	6.7
20–24 years	2	6.7
25–29 years	0	0
30–34 years	1	3.3
35–39 years	0	0
40–44 years	1	3.3

One participant had concurrent controlled epilepsy, eight had a confirmed previous misdiagnosis of epilepsy, and one still felt that his seizures could be a mixture of the two, despite clinical opinion that his diagnosis was only DS. More women than men were interviewed to reflect the higher prevalence of DS among women in the CODES study [22] and the population with DS more widely. The majority (20/22) of participants randomized to receive CBT who were interviewed here attended at least nine CBT sessions and were therefore classified as compliant with the DS-specific CBT in the study [23]. Those interviewees allocated to CBT + SMC attended a median of 3.5 SMC sessions (range: 0–10 sessions), while those patients allocated to SMC-alone attended a median of 4 SMC sessions (range: 2–6 sessions). At the time of their diagnosis by a CODES neurologist, none of the participants reported any previous knowledge of DS.

### 3.2 Themes

Three superordinate themes relating to participating in the trial and treatment emerged from the TFA and are discussed in this paper. The three themes were the following: a) the experience of taking part in a treatment trial — “the only thing out there”, b) treatment techniques that were perceived to help with seizure management, and c) and reflections on an “unpredictable recovery”. The three themes and their subthemes are listed in Table 2.

**Table 2:** Overarching and subthemes arising from the interviews.

Overarching theme	Subthemes
Participating in a treatment trial — the “only thing out there”	a) “The chance of something changing” b) CODES Health Professionals: “People who understand” c) The process of randomization and treatment expectation
Treatment components perceived to be helpful	a) The benefit of written materials — “I thought they were describing me” b) “Finding out a bit more about who I am” — the benefits of a good therapeutic relationship c) “Focusing on my breathing” — techniques that help feelings of control d) Challenges to the implementation of CBT
Reflections on an “unpredictable recovery”	a) Personal insights into DS and attributions for “getting my life back” b) Accepting partial improvement c) Ongoing support

#### 3.2.1 Participating in a treatment trial — “the only thing out there”

##### 3.2.1.1 “The chance of something changing”

Eighty percent of all the interviewees said they consented to take part in the trial because it offered them hope in relation to treatment. Sixty percent recalled how before the trial, they had felt low, desperate, and lost. Common responses in their accounts of why they took part were expressions such as “there's nothing else out there” or “the chance of something changing”, especially for those for whom there had been a query as to whether the seizures were epileptic. Seven participants said that they had also taken part as maybe it would help further research and treatment for others.

##### 3.2.1.2 CODES health professionals — “people who understand”

While many did not comment on this, just over half of all participants from the total of 30 interviewed expressly indicated that they had felt understood by the CODES health professionals (including the research workers who enrolled them into the trial), which in turn, stopped them feeling so alone and isolated:

*“It sort of gets you talking to people and gets you out the house, because you're going to appointments … You know it's not just – you've invented it, that's why you're staying in the house all on your own and it kind of makes you think – right okay it's an actual problem and people are actually dealing with it and other people have it as well”.*

(Female, SMC, interview 29)

Five participants recalled feeling apprehensive about having to see the CODES neuropsychiatrist as they were concerned about the stigma of having a mental illness, attending hospital appointments, and the implications of others finding out:

*“It's just so annoying that the world is so taboo about mental health full stop. So, when you hear about the Psychiatry side…it's like oh God”.*

(Female, CBT + SMC, interview 28)

One participant in the SMC arm recalled liking the psychiatrist but feeling frustrated that he asked too many times about suicide risk and how he felt before a seizure (something he could never remember).

*“That can be a minor frustration for me as well, when people in medical profession say ‘what happens when you have one’ and I say to them ‘I don't know’, you know, I black out I go down next thing I know is I'm coming round couple of minutes later”.*

(Male, SMC, interview 4)

##### 3.2.1.3 The process of randomization and treatment expectation

Almost all participants expressly said that they had understood the rationale of being randomized and that it had been explained to them fully by the CODES team as they moved into the trial; no one specifically indicated that they had not understood the rationale for randomization. There was a range of responses to how participants had felt about being randomized to one of the two treatment arms. In relation to what treatment arm they were randomized to, half of those interviewed said they remembered wanting as much treatment as the trial could offer (i.e., CBT in addition to SMC). However, a further nine recalled feeling hesitant about the possibility of receiving the (additional) psychotherapy and cited a range of obstacles such as how therapy would be painful in terms of “dragging up the past”, that it would be too big a commitment in terms of travel or, incorrectly, that it would mean being in group therapy that they did not want.

Nearly two-thirds of the CBT + SMC participants and just over a third of the SMC participants felt that the experience of the trial had been very positive, citing outcomes such as improved quality of life, seizure reduction, validation by the health professionals of their DS, or their own acceptance of DS:

*“I feel really lucky to have been part of it. I'm the kind of person who is always trying to help other people and do this and do that. When you're in it you're not thinking like that, you're not thinking oh they can use me, it's more like I'm getting all this help and if I hadn't got that diagnosis and that study hadn't been running, don't know where I'd be now. I could still be as bad as I was, so it was a wonderful thing.”*

(Female, CBT + SMC, interview 20)

However, three males and one female participant in the SMC group and one female and one male in the CBT + SMC group felt that while participating in the trial had been largely positive, in terms of the treatment itself, it had either not been enough or not what they wanted. Of these, a male participant in the CBT + SMC group who had previous experience of counseling refused to engage with CBT techniques or complete homework. He dropped out at session 9.

*“I wanted to talk about things and whether they listened was entirely up to them. Just talking about it made me feel better.”*

(Male, CBT + SMC, interview 21)

One female participant, who dropped out of CBT after two sessions, felt frustrated by aspects of the trial. She stopped attending CBT at session 2 because she did not feel emotionally ready to discuss her trauma.

*“Cos normally when I have therapy it's about the future…she (the therapist) was talking about digging up pain. I didn't feel like I was clever enough. I didn't feel like I was emotionally clever enough to be doing that so far away from home as well.”*

(Female, CBT + SMC, interview 15)

She also felt irritated that she could not then disclose to the trial research worker (who phoned her fortnightly for her seizure diary) her reasons for stopping as he would then become unblinded to her treatment arm.

The female participant in the SMC group felt that while she had felt understood initially in the trial, she had wanted more treatment than SMC and had not experienced a positive relationship with the psychiatrist.

*“I feel like every time I see him he's got me so worked up that he wants me to believe this thing (dissociative seizures) but he's given no help or advice to me rather than if I say the wrong word (epilepsy) he's quick to snap and say it's not that.”*

(Female, SMC, interview 1)

#### 3.2.2 Treatment components perceived to be helpful

##### 3.2.2.1 The benefit of written materials — “I thought they were describing me”

Irrespective of the arm to which patients were randomized, all had received written booklets on DS, one at the time of diagnosis by the neurologist and a further one at the psychiatry assessment. Twenty-two trial participants (over two-thirds of the total sample) expressly indicated that, in addition to the explanation provided by the neurologist, the CODES Factsheets offered them the opportunity to further understand their condition and helped them explain the condition to others, including other people involved in their care such as general practitioners:

*“I remember crying I think cos I was just so, finally…“this is what I've got, this is me, this makes sense”. I remember saying “this is me”. To have that there was really helpful. I definitely know that there needs to be so much more about that.”*

(Female, CBT + SMC, interview 3)

Written materials supporting the CBT sessions were also found to be useful for participants reporting poor memory. As one participant reported:

*“So if you have something visual you can feel that you're getting anxious or you can feel that it's a time of stress and you can go to that, pick it up and look at it and go right okay well this is what I did before. Did it work last time? Yes, brilliant. And it's also a way of stepping back because you have to stop and you have to look at it and that in itself can sometimes just be the difference between going overwhelmed and going off the boiling point”*

(Female, CBT + SMC, interview 23)

Nonetheless, not all participants read the booklets in detail, with one in the CBT + SMC arm indicating that they had not wanted to, and one in the SMC alone arm indicating that she had difficulty reading anything in a book format. A further person in the SMC arm felt that they did not provide him with any new information.

##### 3.2.2.2 “Finding out a bit more about who I am” — the benefits of a good therapeutic relationship

Fifteen of the 22 participants who were randomized to receive CBT + SMC described a positive relationship with the CBT therapist and felt that this had been a key component in their improvement. Two further participants talked about the content of the CBT sessions rather than the actual relationship. One of the participants (mentioned in Section 3.2.1.3) who dropped out at the second CBT session said that she had not formed any alliance with the therapist; another person (also quoted in Section 3.2.1.3) described a positive alliance but, as indicated earlier, wanted to keep talking in sessions rather than engage in CBT techniques. While several said they recalled feeling an initial fear of having to talk to someone, their ability to trust the therapist gradually built up over time and, in a few cases, prompted them to newly disclose trauma. Many of the participants recalled feeling that their therapists were extremely skilled at their ability to ask the right questions.

*“(The CBT Therapist) had a way with you to get you to talk about it without even knowing you're doing it”*

(Male, CBT + SMC, interview 11)

*“I looked forward to every meeting, because I had something to share, something progressive, my progress to share and they would always be very encouraging and probably it's not the best word to use but proud. Really happy for me and obviously…they kept saying to me it's what you're doing but obviously it was what they were doing too.”*

(Female, CBT + SMC, interview 17)

There was a range of opinions regarding the SMC sessions and not everyone commented on their relationship with the psychiatrist. Twelve out of the 30 participants across the two treatment arms clearly felt that the SMC sessions with the psychiatrist had been helpful or that they felt listened to. Of the 12, some commented on how the sessions provided a positive and nonjudgmental space in which they could speak at ease about whatever they wanted to discuss. Others commented positively on the fact that the psychiatrist had offered help should they need it in the future. They also reflected how the CODES psychiatrist had reinforced the diagnosis in a way that they believed was beneficial in terms of accepting and understanding it. For the majority of the remainder, there was no specific comment.

The benefits from SMC sessions with the psychiatrist were reflected in different ways. For two participants in the CBT + SMC group, the easy relationship with the psychiatrist allowed them to compliment the sessional work with the CBT therapist and deal with the consequences of disclosing childhood trauma, suggesting some benefit from the multidisciplinary input in the trial. For one male SMC-alone participant, the positive relationship with the psychiatrist, who had been instrumental in making the DS diagnosis prior to his participation in the RCT, was key to his progress in the trial. He felt he could talk openly without feeling judged. He and one other SMC-alone participant reflected how unsure they were that they would have coped with CBT as they felt that the meetings with the psychiatrist were sufficient. However, one SMC-alone female participant said she felt unsupported by both the neurologist and the psychiatrist in her coming to terms with a DS diagnosis after being originally misdiagnosed with epilepsy and having taken antiepilepsy medication for years.

##### 3.2.2.3 “Focusing on my breathing” — techniques that help feelings of control

The disabling physical and psychological impairment brought about by DS combined with the widely varying semiology, both between and within individuals, left many of the participants in this study feeling “out of control”. This absence of control was not just in relation to the seemingly random nature of DS but was exacerbated by their other comorbid health conditions. Participants reported feeling that they had no control not just over seizures but also over their bodies and minds. They described how this feeling was more acute during the often lengthy process of receiving the correct diagnosis. It follows then that the introduction of any perception of control may be important to individuals with DS. CBT therapists introduced participants to a range of techniques and exercises to help in the management of seizures including, but not exclusively, progressive muscle relaxation, breathing, distraction and refocusing, visualization, and graded exposure. Control was discussed in the interviews only in relation to CBT techniques.

Participants in the CBT + SMC group indicated that there were benefits from being able to choose from a range of techniques the ones that suited them best. Eighteen of the 22 CBT + SMC participants found that learning to control and slow down their breath by breathing from the stomach or breathing *through* pain helped them relax and even divert a seizure:

*“She (CBT therapist) said you're getting the anxiety, which is building up as muscle tension, and to release it you need to breathe through it. Um so I breathed through that and once I had released the pain from my stomach, I could then face the problems in front of me.”*

(Female, CBT + SMC, interview 27)

Thirteen CBT + SMC participants who found it helpful said that they continued to use this technique for DS management after treatment had ended. One SMC-alone participant, who had researched techniques online, also found controlled breathing was helpful. Seven of the 22 CBT + SMC participants found that distraction was particularly useful for diverting or preventing seizures, including two people who had found that breathing deeply made them feel light-headed or dizzy.

In the CBT + SMC group, the self-management or control of seizures was often reflected in participants' ability to understand the triggers and to then recognize the warning signs. Increased control was often linked by participants to the start of improvement, and a gradual awareness of warning signs led to some being able to postpone the seizure until they had found a “safe place”. One female participant reflected that she had already been practicing an element of control over the seizures before the trial but talking to the CBT therapist or CODES psychiatrist gave her confirmation that this was the right thing to do. Some, as they recovered, were able to divert or even completely avoid a seizure:

*“I guess on some level I was aware that, it wasn't just going to happen out of the blue, I knew when it was coming and yeah. I always knew, I guess, that I could put it off. But I don't think it was until I started going through the trial and having the CBT that I realised that I could control, I had complete control over that and I could use that to make myself better”*

(Female, CBT + SMC, interview 24)

For six of the 22 receiving CBT + SMC, having more control over a seizure meant that allowing one to happen was more a “conscious decision”. However, there were discordant views about participants' perceived ability to control seizures. Not everyone felt that they had warning signs. One participant who had disengaged from CBT towards the end felt learning any techniques would be pointless as he had no warning signs of a seizure. He reported, however, that his wife could tell by his changed facial expression that he was about to have one.

##### 3.2.2.4 Challenges to the implementation of CBT

One of the challenges that patients reported as part of CBT was being able to engage with the tasks, often because of emotional or behavioral avoidance. Two male participants recalled how they did not want to engage with the techniques and the homework materials given to them by their CBT therapists because they could not see the value of either. Another female CBT participant felt that she was unable to write down her emotions as part of her homework as this was too distressing to do outside of therapy sessions.

For one woman in the CBT + SMC group who had been frequently housebound and had high levels of social anxiety, behavioral experiments such as walking into town proved very difficult. She recalled her fear of attempting behavioral experiments such as walking around in public with the therapist at her side:

*“And we walked from the hospital to the town and that was just, yeah, mental. I actually feel like I could have strangled her (the therapist)…You're not just frightened of the seizure but what other people are going to do. Are they going to hurt you, are they going to kick you? … And are cars going to run you over and are people going to look at you?”*

(Female, CBT + SMC, interview 13)

#### 3.2.3 Reflections on an “unpredictable recovery”

As a number of participants in both the CBT + SMC and SMC-alone arms reported that their interventions helped them to improve seizure management and (in one of the SMC participants and six of the 22 CBT + SMC cases interviewed here) to become seizure-free at the time of interview, participants' insights into how and why they believed that they had changed over the course of treatment gave rise to a further theme. The process of recovery, as perceived by a number of participants, was neither linear nor straightforward. They felt that feeling better was not just about seizure reduction but an acceptance of the seizures – or at least recognizing that they did not have to be scared of them – and an acknowledgment, and acceptance for some, of their own personality traits or temperament.

*“I think another thing was it was acceptance, acceptance of my medical condition and acceptance of seizures, because I think up until that point I found it very difficult to accept.”*

(Male, CBT + SMC, interview 12)

##### 3.2.3.1 Personal insights into DS and attributions for “getting my life back”

There was a wide variation in participants' accounts of the process of improvement. Eleven participants were able to reflect openly on a range of factors, both external and internal, that they believed had left them prone to developing DS in the first place. They often recalled very difficult events such as the death of a parent at a young age, relationship disturbances, mental health problems, or comorbidities such as fibromyalgia, or the potentially life-threatening illness of a child. However, there was also insight into their own characteristics or types of personality that they also thought had predisposed them to stress. Three said they now understood how they had previously been more emotionally cutoff or disconnected from their own emotions while realizing they had put too much emphasis on others' emotional cues and responses.

In turn, this led almost half of the interviewed CBT + SMC group and a quarter of the interviewed SMC-alone group to describe a type of emotional shift, a process that they viewed as mainly positive and, by their own understanding, interlinked with improvement. One participant described CBT being the conduit to being open to feeling more extreme emotions rather than living life in an emotional “gray middle”, which he understood had been a useful coping strategy:

*“I'm getting closer to that full range of feeling, which is good but it's bad because… I guess it means that whatever I was doing in the past was working in that I was obviously trying to avoid the extremes, so what I was doing was working, my coping strategies, my coping mechanisms, my routines were serving a purpose”*

(Male, SMC-alone, interview 22)

Several CBT + SMC participants described how therapy had encouraged them to feel less overwhelmed in their response to stress and being either more vocal about their needs and emotions or more consciously prioritizing them over the needs of others. Just over a third of the CBT + SMC and a quarter of the SMC-alone interviewees had come to realize how much anxiety, worry, anger, and frustration they had been holding on to over the years, which had been highlighted in sessions either with the CBT therapist or the psychiatrist. One CBT + SMC participant described how she had become more openly emotional and how therapy had shown her the importance of being able to cry in front of others. For another, having CBT was the catalyst for having the courage to separate from her partner, and a third CBT + SMC participant felt that therapy had enabled her to speak up for herself in her current relationship. Conversely, a fourth participant receiving CBT expressed her bewilderment that feeling any positive or negative emotion continued to be sufficient in itself to trigger a seizure. A participant in the SMC-alone arm felt that it was the diagnosis itself, which had been the turning point or validation, that allowed him to be more honest with others about his own emotional state:

*“Whereas getting that diagnosis felt I could probably be a bit more, a bit more, not much, a bit more open…about, ‘Look I'm not feeling great today.’”*

(Male, SMC-alone, interview 10)

However, not everyone felt any significant emotional shift. For one female participant in the CBT + SMC arm (who was seizure-free at the point of interview), therapy made her aware of the very high expectations she continually set herself, but she was not able to change this within the course of treatment.

Despite the diagnostic approach and how it was communicated in the study, there were variations in participants' attributions of the causes of their seizures. For three participants, the reduction in seizures was attributed by them to not only receiving the correct diagnosis but also to external factors such as antidepressants (SMC participant), a change in heart medication, which the participant felt stopped her falling (SMC participant), that in turn led her to a complete cessation of seizures and, in the third case (CBT + SMC participant), a sleep apnea machine that the participant said had enabled her to sleep properly for the first time in a long time.

##### 3.2.3.2 Accepting partial improvement

Ten participants across the two treatment arms reflected on their acceptance that progress and improvement may be partial or as unpredictable as the seizures themselves. Coming to terms with the random nature of DS was viewed by a few as a vital component of the process of change. Three participants also recognized that feeling better was something that had to be worked at and that becoming seizure-free or improving seizure management could not be taken for granted.

*“I am still on the path to success for the dissociative seizures, because it's a long road and that road has got a few bumps in it [laughs]. While I have the bumps, particularly because I've had a few bumps soon after I finished the CBT and so it's not that I question whether the CBT has worked, I know it's worked, but it's a question of whether I've got my act together enough, or whether I've been lazy and not been continuing the work after the sessions.”*

(Male, CBT + SMC, interview 12)

Acceptance of DS unpredictability was linked to improvement and equally to being able to reconnect to the enjoyments of life prior to DS:

*“It's thinking about it differently and if I do have a seizure, that's okay, it's not the end of the world whereas before it did feel like this is it, this is my life, it's never going to get better, I can't stand it, it's very, very debilitating. It's not anyway nearly as debilitating as it was.”*

(Female, CBT + SMC, interview 23)

##### 3.2.3.3 Ongoing support

Seven of the eight participants in the CBT + SMC group who were seizure-free at the time of interview felt there was no need for further psychological input. However, a further eight participants in the CBT + SMC group and one in the SMC alone group, who had experienced a reduction in seizures, felt that they needed further support and said they wanted to embark on further therapy. One CBT + SMC participant who had been seizure-free for most of the treatment trial felt that starting a part-time job had triggered a small number of seizures, which (at the point of interview, i.e., after the end of the trial) had prompted her to consider the possibility of accessing CBT privately.

Avoidance of traumatic memories characterized some of our participants prior to therapy. Nineteen of the interviewees in this study spoke explicitly or implicitly about some sort of perceived trauma. For 14 of the CBT + SMC participants and three of the SMC participants, having a therapist or psychiatrist that they could talk to was described as a turning point in terms of feeling able to trust someone enough to disclose previously undisclosed – and what were, for some – traumatic events. Two women in the CBT + SMC arm said the powerful emotions and increase in seizures accompanying these recollections were overwhelming and almost derailed therapy. They felt they needed ongoing trauma-focused therapy after CODES support had ended despite having a very positive therapeutic alliance with the psychiatrist as well:

*“It is like a sleeping dragon that has been poked and prodded and is now roaring and affecting everything, absolutely everything, and I'm left with nothing. Worse than nothing really because it's awoken, and it won't get back to sleep.”*

(Female, CBT + SMC, interview 23)

## 4 Discussion

The aim of our qualitative study was to explore the experiences of participants with DS who had taken part in an RCT comparing SMC-alone with DS-specific CBT in addition to SMC. The CODES study employed a specific care pathway involving neurologists and psychiatrists (plus CBT for those allocated to receive it) and a range of trial-specific components [12]. Gordon [24] highlighted the importance of researching the client's view of psychotherapy. Examining these issues qualitatively enriches the understanding of what treatment techniques might help improve seizure management but also throws light on what might underlie lack of treatment success or what might make the treatment process less smooth.

The first theme discussed here was the experience of taking part in a trial. To our knowledge, this is the first qualitative study to ask people with DS whether taking part in a treatment trial had been acceptable. Participants said they understood the need for randomization and that it had been fully explained. Only half of the participants had wanted as much treatment as might be on offer (CBT + SMC), as many recalled feeling a mixture of apprehension or skepticism about having 12 CBT sessions; future treatment studies should bear such apprehension in mind when discussing trial requirements with participants prior to enrolment. For the majority, trial participation had resulted from having no other treatment options available or offered to them. For just over half of those interviewed here, taking part in the trial had been of value, and treatment had led to improvement, i.e., either a reduction in or discontinuation of seizures, while for others, it meant an improvement in their acceptance of the seizures. Most participants had felt supported by the health professionals in the study. A majority of interviewees felt that the written materials in booklet form were very helpful as they could re-read them in their own time and provide information to explain DS to others.

The second theme was the relative helpfulness of CBT techniques in seizure management. In terms of reflections regarding seizure management, having an element of control over seizures was seen as crucial to any path towards positive change. The clear majority of CBT + SMC interviewees felt that breathing and distraction techniques learnt during the study had allowed them to gain some control over the seizures and, with it, the disruption of previous cycles of avoidance. Indeed, such techniques have been recommended elsewhere for managing DS [25]. Most of the participants interviewed here reported that they had been exposed to a range of CBT techniques that could be practiced easily (and on an ongoing basis) to prevent or interrupt behavioral/cognitive/emotional chain of events in DS. These were perceived to have been crucial to their seizure management and, for some, their path to improvement. Previous research has highlighted the sense of powerlessness and uncontrollability [26] felt among people with DS as well as an external locus of control. It is encouraging, therefore, that so many participants reported continuing to use these techniques several months after therapy had stopped.

The importance of the therapeutic relationship between the patient and both the therapist and psychiatrist reported here supports previous research concerning the relationship between a positive therapeutic alliance and treatment outcomes in psychotherapy [27]. The present study showed that participants in both treatment arms believed that having a positive therapeutic alliance (either with the CBT therapist or with the psychiatrist or both) had contributed towards them becoming more aware and connected to their emotions and even more expressive of them in front of others.

Emotional avoidance on the part of our participants posed challenges to the implementation of CBT techniques, despite being identified as important for therapists to address. Several studies have previously highlighted the role of emotional avoidance, disconnectedness, or alexithymia in people with DS [16,[28], [29], [30], [31]]. More specifically, Cullingham et al. [32] found higher levels of experiential avoidance (a broad range of avoidance encompassing both behavioral and cognitive strategies that are used to avoid distressing or difficult personal experiences as a result of fear of those experiences) in people with DS when compared with those with epilepsy or healthy controls. We have also recently shown [33] that our overall sample of patients with DS entering the RCT had high levels of belief in the unacceptability of experiencing or expressing negative emotions. Our qualitative study builds on these previous studies and showed some evidence that CBT had allowed some participants to engage more fully with their emotions (a change that they viewed as positive) and to be less fearful of the consequences of doing so. This is of particular clinical relevance and needs further consideration in future treatment trials, since it has been proposed that the avoidance of emotions may be one factor that perpetuates the difficulties associated with poorer quality of life in people with DS [34]. We further speculate that difficulty in emotional expression and in recognizing the associations between emotions and seizure occurrence may be one factor common to those patients who may have found treatment difficult, and this poses further challenges for those providing therapy for this patient group.

Participants, particularly in the CBT + SMC group, felt that improvement was not just linked to seizure management but also to the acceptance of the apparently unpredictable nature of the seizures themselves, in particular for individuals with no specific warning signs or possibly those who had no recollection of their seizures or preceding events. Of potential relevance here, Cope et al. [35] believe that there is evidence for using third wave CBT, acceptance and commitment therapy (ACT) in the treatment of DS. While the DS-specific CBT used in the CODES trial was more traditional (focusing on challenging and changing thoughts and beliefs), ACT focuses on altering the person's relationship to their thoughts, feelings, and physical experiences. However, if therapists consider offering patients ACT or other models of psychotherapy, the evidence-base should be taken into account. It is important to note that for interventions other than the DS-specific CBT evaluated in the CODES trial, evidence is not available from adequately powered multicenter RCTs. In addition, the specific effects of some third wave interventions such as mindfulness on the experience of dissociative phenomena are unknown.

Our findings suggest that the course of improvement was variable for participants in both groups. Being protocol-driven, the number and content of the CBT sessions was predetermined, but it was recognized that, at the end of the study, additional interventions might be warranted, although not for all participants. For two of the participants in the CBT + SMC group, the emotional processing of trauma in therapy meant that they had become more unwell or had got worse before they got better. Although a temporary worsening of symptoms is common when treating patients, our findings attest to the importance of ongoing work by the individual to manage the condition after therapy ends, i.e., using CBT techniques long after therapy in the trial had been completed. For other participants, fewer sessions may have been sufficient, and it is possible that outcome may not be directly related to the number of sessions attended, suggesting that to some extent at least, individual tailoring of the length of treatment may be helpful.

### 4.1 Strengths and limitations of this study

Although previous qualitative analyses have described reactions to therapy [13], our study focuses in depth on the experience of receiving DS-specific CBT and accompanying SMC. This study will ultimately complement our quantitative analysis from the CODES study.

One of the limitations of this study is the potential for respondent bias. In some cases, research workers carrying out the interviews were known to participants as they had collected outcome data from them during the study. This could have led participants to feel pressured to be less critical of the study. Conversely, participants may have felt sufficiently comfortable with the research worker to disclose difficult information. A further limitation is that one of the CBT + SMC participants did not receive any follow-up sessions in SMC and so was unable to comment on any experience of SMC components during the interview. In addition, all our participants had met the trial's eligibility criteria [12] and had been willing to accept referral to a psychiatrist and take part in CBT if allocated to that treatment arm, and take part in this qualitative study, potentially making them less representative of people with DS overall. The ethnic composition of the sample was heavily biased towards white British, although this reflects the composition of the wider population from which the RCT sample was drawn [22]. Nonetheless, other cultural backgrounds might give rise to the need to address different beliefs about DS and the contexts in which they might occur [36], and might affect whether patients attend treatment sessions. In addition, the specific care pathway and protocol followed in the CODES study may have led participants to be better informed about their condition by the time they met with their psychiatrists and commenced CBT than might typically be the case. Finally, while we believe that we reached a saturation and consistency of themes in relation to our CBT + SMC participants, we cannot be certain that interviewing a larger number of SMC participants would not have yielded more themes, despite our best efforts to capture diversity through purposive sampling.

## 5 Conclusions

Participants in this study were a diverse, heterogeneous group of individuals. The findings show the feasibility and acceptability among people with DS of taking part in an RCT offering DS-specific CBT from therapists and specialist medical care from neurologists and psychiatrists. Participants in the CBT + SMC arm of the trial found breathing techniques and distraction to be very important in helping with seizure management and control. Positive change was not always a matter of becoming seizure-free but included finding ways of managing DS occurrence.

## Funding statement

This paper describes independent research funded by the 10.13039/501100000272National Institute for Health Research (Health Technology Assessment programme, 12/26/01, COgnitive behavioral therapy vs standardized medical care for adults with Dissociative non-Epileptic Seizures: A multicenter randomized controlled trial (CODES)). This paper also describes independent research part-funded (LHG, TC) by the National Institute for Health Research (NIHR) Maudsley Biomedical Research Centre at the 10.13039/100009362South London and Maudsley NHS Foundation Trust and 10.13039/501100000764King's College London. JS is supported by an NHS Scotland NHS Research Scotland (NRS) Career Fellowship and the CODES research team also acknowledges the financial support of NRS through the Edinburgh Clinical Research Facility. The CODES study benefitted from the support of the NIHR Sheffield Biomedical Research Centre (Translational Neuroscience). The views expressed in this publication are those of the authors and not necessarily those of the NHS, the NIHR or the Department of Health and Social Care.

## Declaration of competing interest

Markus Reuber is the paid Editor-in-Chief of Seizure-European Journal of Epilepsy and receives authorship fees from Oxford University Press in relation to a number of books about dissociative seizures. Jon Stone reports independent expert testimony work for personal injury and medical negligence claims, royalties from UpToDate for articles on functional neurological disorder and runs a free nonprofit self-help website, www.neurosymptoms.org. The remaining authors have no conflict of interest to declare.
